# Effect of process variables on optimisation of mechanical properties of silica/poly(tetrahydrofuran) sol-gel hybrids

**DOI:** 10.1007/s10971-025-06725-5

**Published:** 2025-03-22

**Authors:** Yu-Chien Lin, Gloria Young, Haffsah Iqbal, Achintha I. Kondarage, Angelo Karunaratne, Julian R. Jones, Francesca Tallia

**Affiliations:** 1 Department of Materials, Imperial College London, South Kensington Campus, London, UK; 2 Department of Mechanical Engineering, University of Moratuwa, Moratuwa, Sri Lanka

**Keywords:** Inorganic/organic hybrid, Mechanical properties, Sol-gel, Cationic ring opening polymerisation

## Abstract

Inorganic/organic sol-gel hybrid materials consist of inorganic and organic co-networks which interact at the molecular level and behave as a single material. The aim is to gain synergy of properties between the two components, with the inorganic providing mechanical strength and organic providing elasticity. This becomes more challenging as the thickness of a sol-gel material increases, due to risk of cracking. Here, we investigate the effect of sol-gel processing variables for large silica/ poly(tetrahydrofuran) (SiO_2_-PolyTHF) monolithic hybrids (a size range of several centimetres) to optimise their mechanical properties. Cracking on drying can occur due to combinations of shrinkage and capillary stresses inside the monoliths, but can be mitigated by optimisation of process variables and hybrid composition. The effect of these variables on success rate of producing crack-free monoliths was assessed, then full characterisation carried out on samples made with successful protocols. Computational scribe calculations on X-ray micro-computed tomography images determined the shrinkage during different drying methods. Chemical, thermal, mechanical, and structural analyses were applied to devise a reproducible synthesis method for hybrid monoliths. The monolithic hybrids, prepared using both oven-dried (OD) and freeze-dried (FD) techniques, demonstrated compressive strengths of 44.1 MPa and 30.5 MPa, respectively, when made with an inorganic content of ~31 wt.%. These materials can withstand strains of up to 54.5%. Mechanical testing in wet and dry conditions, i.e. after 60 days immersion in phosphate buffered saline solution (PBS), enabled observation of the viscoelastic response after 1000 cycle compression loading, demonstrating that the materials can work under wet conditions.

## Graphical Abstract

**Figure:**
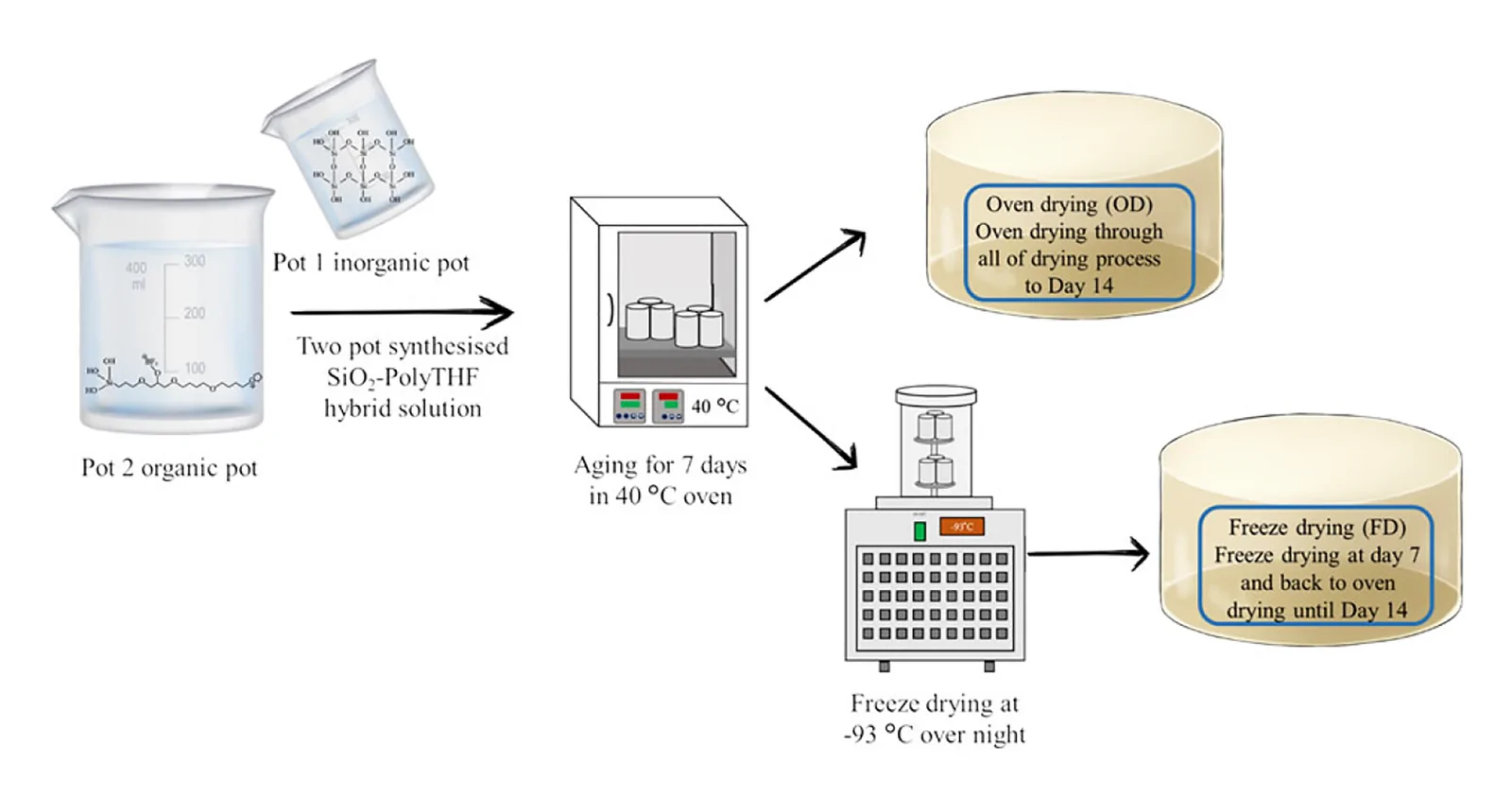
Production of large monolithic tough inorganic/organic class IV hybrid materials through optimisation of sol-gel and drying processing with in situ cationic ring opening polymerisation (CROP) of poly(tetrahydrofuran).

## 1 Introduction

Hybrid materials are distinguished from composite materials in that the inorganic and organic components interact at the molecular scale as co-networks, which can be bonded using coupling agents [1–3], to improve the likelihood of gaining true synergy of the properties of the inorganic and organic moieties [4]. The inorganic, often silica (SiO_2_), provides stiffness, while the organic polymer should impart elasticity and toughness. Silica-based hybrid synthesis is usually by addition of a polymer into a tetraethyl orthosilicate (TEOS)-based sol-gel process at room temperature, prior to gelation and drying. Simple addition of a polymer would create a class I hybrid [5], wherein the only bonds between the networks are physical bonds, such as hydrogen bonds, which can lead to instability on ingress of water [6]. If the polymer is functionalised with organosilane groups prior to its introduction to the sol, using a coupling agent, such as 3-glycidoxypropyltrimethoxysilane (GPTMS) [6, 7] or 3-isocyanatopropyl triethoxysilane (ICPTS) [8], the-SiOR groups can hydrolyse and the resulting -SiOH groups can undergo polycondensation with the silicate network, creating a class II hybrid that has covalent bonds between the inorganic and organic networks. Examples of class II hybrids made with biodegradable synthetic polymers include silica/ poly(ε-caprolactone), wherein poly(ε-caprolactone) (PCL) diol was functionalised with ICPTS before being introduced to the hydrolysed TEOS sol [9, 10]. Silica/PCL hybrids with 60 wt% PCL (molar mass ~6.7 kDa) had a Young’s modulus of 582 MPa and a tensile strength of 21 MPa. Further improvement of properties was limited because the coupling site was only at the ends of the polymer chains and so coupling could not be changed independently of polymer molar mass.

Application of silica-based sol-gel hybrids is often limited to coatings, thin films and 3D printing [11, 12] due to the large degree of shrinkage and risk of cracking during drying of larger monolithic materials [11, 13]. Crack formation during hybrid synthesis can be caused by capillary stresses during drying, exacerbated by phase separation. Phase separation is likely to be affected by: solvent type and the speed of evaporation of the solvent; chemistry of the organic component (e.g. hydrophilicity and presence of-SiOH); temperature; pH of the acidic sol-gel catalyst; and inorganic/organic (I/O) precursor ratio. As the organic content increases, the rate of phase separation reduces,while the rate of sol-gel transition remains unchanged. This causes gelation to take place during the initial polymerisation process, resulting in the formation of a fine silica skeleton [14]. Reducing phase separation is crucial to investigate the effect of the I/O ratio on success rate of producing homogenous monoliths.

Examples of other silica-based sol-gel hybrids with covalent bonding between networks that avoid phase separation include superhydrophobic silica-polydimethylsiloxane (PDMS) [15] and silica-methacrylate (MA) hybrids [16], where coupling sites are already “built into” the polymer chains. Methacrylate polymers can be synthesised with 3-(trimethoxysilyl)propyl methacrylate (TMSPMA) units that provide -Si-OCH_3_ sites to the chains [17]. Silica-MA hybrids can enhance corrosion resistance, provide surfaces for cell attachment [18] and hydroxyapatite precipitation [19] and can be 3D printed into porous structures with high mechanical strength and strain to failure [20]. However, they are not inherently biodegradable [21].

Tallia et al. [12] developed a new type of class IV hybrid [2] (in situ polymerisation of the organic network and formation of covalent bonds between the co-networks) based on poly(ε-caprolactone)-diol. The hybrid formed through in situ cationic ring-opening polymerisation (CROP) within the sol to produce silica/poly(tetrahydrofuran)/poly(ε-caprolactone) hybrid (SiO_2_/PolyTHF/PCL-diCOOH) materials, which exhibited excellent mechanical properties, elastomeric behaviour and intrinsic autonomous self-healing ability. This combination of sol-gel and in situ CROP benefits crack-free synthesis by reducing phase separation and cracking. GPTMS was used as the coupling agent to bridge inorganic (hydrolysed TEOS) and oxidised PCL (PCL-diCOOH), but the epoxide ring of the GPTMS also acted as an initiator for tetrahydrofuran (THF) poly-merisation, resulting in in situ CROP to PolyTHF [12], reducing the risk of phase separation. These SiO_2_/PolyTHF/ PCL-diCOOH hybrids were biodegradable due to the presence of PCL-diCOOH. A variation to this system included the addition of titania in the inorganic component to increase the strength [22].

Young et al. [23] produced non-degradable hybrids, silica-poly(tetrahydrofuran) (SiO_2_-PolyTHF) with covalent coupling, by eliminating PCL-diCOOH from the process. Proof-of-principle studies showed SiO_2_-PolyTHF hybrids exhibited an unprecedented range of mechanical properties, ranging from elastomeric to glassy, with stiffness between 2 and 200 MPa at 10% strain depending on silica content. At 9 wt.% SiO_2_, hybrids had a failure stress in compression of 8 MPa at 70% strain, and in tension of 2 MPa at 80% strain [23]. Fan et al. further demonstrated that SiO_2_-PolyTHF hybrids combine strong and tough mechanical properties, thanks to the simultaneous tailoring of the I/O ratio (that controls strength) and the size of silica nanoparticles (that influences the number of non-covalent interactions, which provide toughness [24]). These studies have indicated that SiO_2_-PolyTHF hybrids can alter their properties by adjusting the SiO_2_ content [23, 24]. In this first example of SiO_2_-PolyTHF system, excess THF was always used, acting both as solvent and monomer. However, excessive solvent leads to more shrinkage during drying, which affects the successful synthesis of bulk samples.

This study systematically explores how the I/O ratio influences shrinkage, polymerisation behaviour, structural integration, and mechanical properties. Examining the effects of the I/O ratio in the SiO_2_-PolyTHF hybrid system provides valuable insights into optimising synthesis conditions and tailoring the physical properties of hybrids for various applications. The drying process also plays a critical role in the formation of the overall structure. Oven drying (OD), due to solvent evaporation in an open environment, often alters intermolecular forces, such as van der Waals forces, leading to structural deformation. In contrast, freeze drying (FD) can mitigate this issue by inhibiting dehydration-induced damage to the structure [25] and, in some cases, maintaining the molecular structure in a colloidal state during drying [26]. This method allows materials to retain their morphology when combined with other materials to form composites. FD has previously been employed to produce 3D porous scaffolds made of silica/ chitosan hybrids using GPTMS as a coupling agent and water as a solvent [27]. However, this technique has never been applied with an organic solvent such as THF. Consequently, the FD method was selected to evaluate whether bulk samples could achieve comparable or acceptable structural and physical properties.

Therefore, the goal of this investigation was to optimise the sol-gel process variables, including the volume of THF and drying methods and their effect on the shrinkage, for the production of large SiO_2_-PolyTHF monoliths. These monoliths were designed to have a size range of several centimetres and to exhibit high compressive strength and strain to failure, reflecting a high level of toughness. The synthesis was evaluated by observation of phase separation, surface and fracture surface morphologies, and mechanical testing in compression. To further explore the potential applications of SiO_2_-PolyTHF, the materials were immersed in phosphate-buffered saline (PBS) for 60 days, followed by cyclic loading tests under both dry and wet conditions. This simplified simulation of in vivo conditions aimed to verify whether prolonged contact with PBS would cause structural degradation and performance deterioration. These findings and their implications are thoroughly discussed in this study.

## 2 Materials and methods

### 2.1 Materials

All chemicals were purchased from Sigma Aldrich (Dorset, UK). Tetrahydrofuran (THF), (3-glycidoxypropyl) trimethoxysilane (GPTMS), boron trifluoride diethyl etherate (BF_3_·OEt_2_), tetraethylorthosilicate (TEOS), hydrochloric acid (1 M HCl), PBS solution were used directly without further purification.

### 2.2 Hybrid synthesis

I/O SiO_2_-PolyTHF hybrids synthesis was based on the twopot synthesis developed by Young et al. [23], but here, effect of molar ratios of key reagents were investigated, as listed in Table 1. The inorganic pot (Pot 1) was prepared by hydrolysis of TEOS in 1 M HCl and deionised water with a 1:3 vol% ratio in a sealed beaker stirring at 400 rpm for at least 1 h, during which the sol went from cloudy (phase separation) to clear. The organic pot of THF with GPTMS (Pot 2) was stirred at 400 rpm for at least 15 min with molar ratios THF:GPTMS of 25:1, 50:1, 75:1, and 100:1 in a sealed perfluoroalkoxy (PFA) beaker, respectively. The in situ CROP of THF monomer was initiated by the following addition of BF_3_·OEt_2_ catalyst. Bubbles formed in Pot 2 after a few minutes of mixing, due to increase in viscosity, at which point the hydrolysed TEOS sol from Pot 1 was added dropwise, maintaining stirring at 400 rpm for 10 min and then left to stand without stirring for 10 min to allow bubbles to escape from the sol. Mixing times are important for optimisation as the in situ CROP is quenched when the hydrolysed TEOS is mixed dropwise as soon as bubbles formed. Otherwise, rapid polymerisation occurs before the silica network can integrate with the polymer, if TEOS is added too late.

The I/O sols were poured into cylindrical polytetrafluoroethylene (PTFE) moulds of 15 mm in diameter, 25 mm in height. The moulds were sealed inside poly(-methyl pentene) (PMP) pots with screw tops that were also sealed by parafilm and placed in an oven at 40 °C for 7 days. These 7 days represent the ageing time, which ensured the polymerisation completion and minimised THF evaporation. At day 7, two different drying processes were employed. For oven-dried (OD) samples, the screw top lids were opened gradually over a 3-day period to completely open to allow gradual evaporation of THF and then the samples were allowed to dry in open moulds in an oven at 40 °C for further 3 days. Another group of samples were freeze-dried (FD), for which the monoliths were put into a −80 °C freezer for at least 4 h to freeze samples and the lid was open as samples were placed in the freeze drier, which was operated at −93 °C overnight. It should be noted that this is not true FD for these materials, as the melting point of THF is −108.4 °C. However, the freeze drier does provide a different drying procedure to remove solvent more rapidly than OD, with the aid of the vacuum pump. After the FD process, samples were allowed to equilibrate to room temperature and were placed back in the 40 °C oven, the rest of steps were the same as OD condition. Samples are named after their molecular ratio of THF:GPTMS and the drying methods, i.e. 25OD, 25FD, 50OD, 50FD, 75OD, 75FD, 100OD, and 100FD. For instance, sample 25OD means a sample with THF:GPTMS molar ratio of 25:1 and was oven dried.

### 2.3 Solution state NMR

**Fig. 1:**
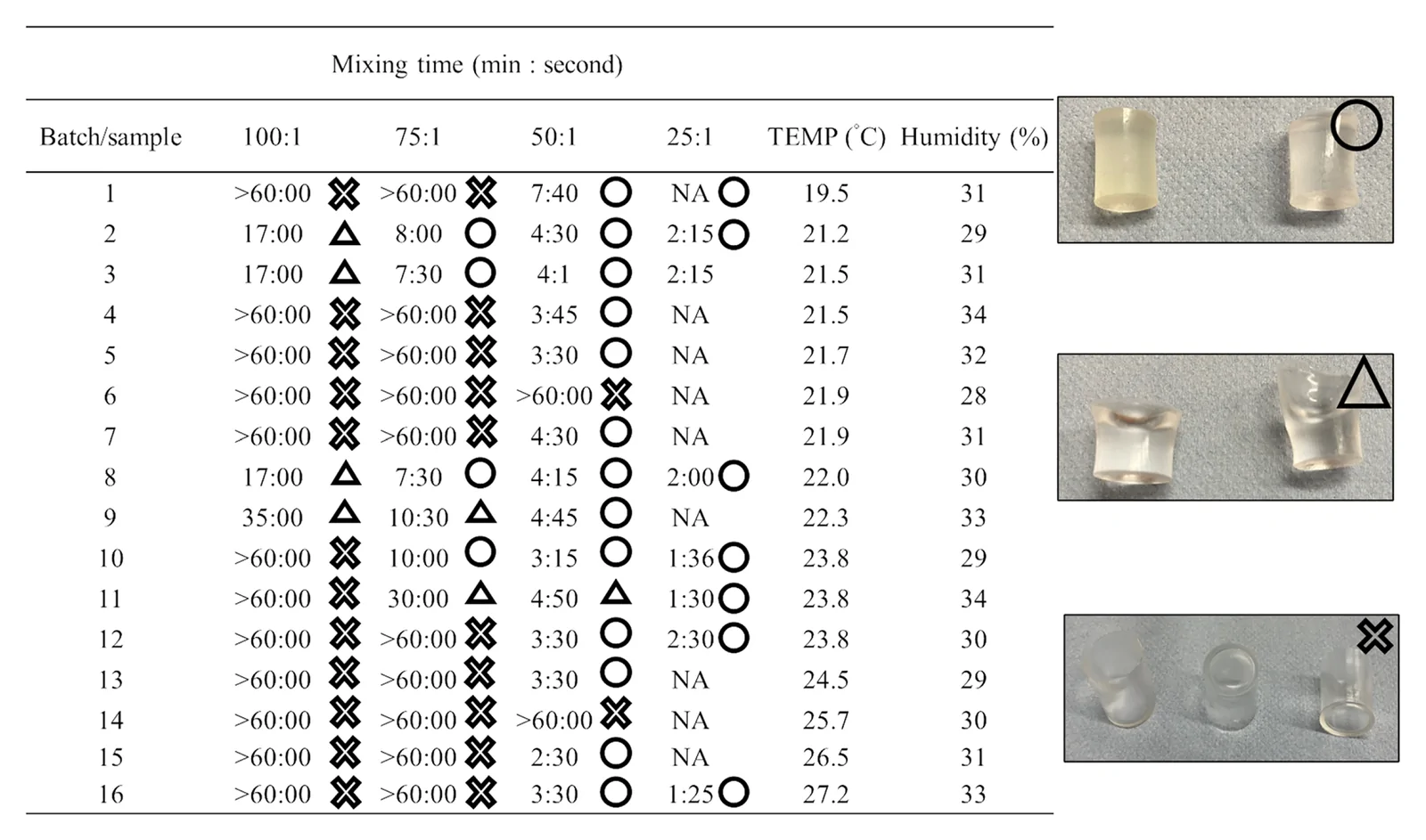
Investigation of effect of THF:GPTMS ratio and mixing time, before beginning dropwise addition of Pot 1 to Pot 2 under oven-dried (OD) conditions, on the morphology of SiO_2_-PolyTHF cylinders. Photographs show samples were either homogeneous cylinders (black circle); distorted cylinders (black triangle) or hollow cylinders (black X) (Cylinder diameters were 1.2 cm; NA not attempted)

Nuclear magnetic resonance (NMR) spectroscopy of hydrogen (^1^H atoms) was used to confirm the opening of the GPTMS epoxide ring and the formation of PolyTHF after mixing with BF_3_·OEt_2_ catalyst, which together indicate the formation of a polymer network. ^1^H NMR (DMX 400, Bruker, USA) was carried out in CDCl_3_ solvent at 400 MHz. The NMR analysis parameters were set to take 16 scans in the range of chemical shift 0–12 ppm. Samples of SiO_2_-PolyTHF hybrid solution were collected for NMR before and after the bubble formation and one drop added into CDCl_3_ to terminate the reaction at each time point until the solution became too viscous to collect, steps are shown in Fig. 1. The analysis was done before the addition of the TEOS solution to confirm the formation of PolyTHF and to study the relationship between the increase in molecular weight and bubble formation. An important step was to verify which time point is more suitable for the addition of TEOS for hybrid synthesis.

### 2.4 μCT and shrinkage factor determination

Shrinkage factor was determined by geometrical measurements and computational script calculation results based on X-ray microtomography (µCT) imaging. Hybrid monoliths were imaged using a laboratory-based µCT system (ZEISS Xradia 510 Versa). The X-ray source was operated at 70 kV and 75 µA during the imaging. Captured projections were reconstructed using a filtered back projection algorithm resulting in a 3D image matrix of 250 × 256 × 252 with an isotropic voxel size of 19.3 µm. Hybrid samples within the 3D images were segmented using a random forest voxel classifier (a machine learning algorithm) implemented using Ilastik software [28]. Each material composition was scanned in three repetitions. Each sample within the 3D images was labelled, and the volume of those samples (V) was calculated using 3D ImageJ suite plugin of ImageJ software [29, 30]. The shrinkage was then calculated, knowing that the volume of the mould (V_o_) used to produce sample is 4417.86 mm^3^.

### 2.5 Material characterisations

The inorganic content was measured using Thermogravimetric analysis (TGA) on Netzsch Jupiter STA 449C with Proteus software. Hybrid samples were prepared for analysis after drying by manually grinding them with a pestle and mortar and 10–15 mg of hybrid powder was added to a platinum crucible. The TGA analysis was conducted to 800 °C with 10 °C/min heating rate. As the temperature increased, the organic part of hybrid was burnt out leaving the residual silica, assumed to be the weight percentage inorganic in the hybrid. Fourier Transform Infrared Spectroscopy (FTIR) (Thermo Scientific Nicolet iS10) was used to evaluate the chemical structure of hybrid materials with an absorbance module with 32 scans and scan range from 400 to 4000 cm^−1^. Uniaxial compression and cyclic loading tests were conducted using a Zwick/Roell testing machine equipped with a 10 kN load cell, with a compression rate of 0.1 mm/min until failure for the compression test. The strain applied for the cyclic loading test was 20% of the deformation at failure obtained from the compression test. The frequency for each cyclic loading was 1 cycle/s. Samples were polished to 1 cm diameter and 2 cm height cylinders. All mechanical testing was performed under 9 N preload (*n* = 5). A Zeiss Auriga focused ion beam-scanning electron microscope (FIB-SEM) equipped with an Energy-dispersive X-ray spectroscopy (EDS) detector was used at 5 kV in inlens mode, with a working distance of 5–10 mm. To examine the fracture surface morphology of the materials during mechanical testing, samples were prepared for SEM by mounting on aluminium stubs with carbon tape followed by coating with 20 nm of gold layer (Q150T sputter coated, Quorum Technologies, UK) to prevent charging of the sample.

### 2.6 Behaviour in wet environment

To investigate the long-term behaviour of the hybrids in a wet environment, weight loss measurements and cyclic loading test were performed. For the weight loss, SiO_2_-PolyTHF hybrid cylinders polished to 1 cm diameter and 1 cm height were immersed in 10 mL of PBS. Dissolution vessels were placed in an incubating orbital shaker held at 37 °C, and agitated at 120 rpm. The sample mass was recorded after OD overnight at 60 °C and dried mass recorded after for 0, 1, 7, 14, and 60 days. For mechanical testing, cylindrical samples polished to 1 cm diameter and 2 cm height were soaked in PBS for 0 (i.e., presoaking), 1, 7, 14, and 60 days in the incubator at 37 °C with 120 rpm shaking, respectively and recorded 1000 cycle loading test at every time point. Testing was performed under 9 N preload (*n* = 5), with the same samples tested at each time point to monitor the variation in the mechanical response. Samples were compressed at a frequency of 1 cycle/s to 20% of the deformation at failure recorded before soaking.

## 3 Results and discussion

### 3.1 Optimisation of hybrid synthesis

Cylindrical monoliths were successfully produced only under certain conditions. The optimal time for adding inorganic Pot 1 (TEOS hydrolysis) dropwise to organic Pot 2 (organic solution) was at the point of bubble formation in Pot 2, as the polymerisation of the PolyTHF progressed but viscosity was still low enough to allow mixing. Figure 1 shows the effect of THF:GPTMS molecular ratio (100:1 to 25:1) and time of addition of Pot 1 to Pot 2 (after BF_3_·OEt_2_ addition) on the morphology of hybrid monoliths produced. After 10 min of mixing, the solution was cast in PTFE cylindrical moulds of 15 mm in diameter and 25 mm in height. After OD, the monoliths were either homogeneous cylinders (success); distorted cylinders; or hollow cylinders, as shown in the photographs in Fig. 1. A high success rate for producing homogeneous cylindrical monoliths was achieved when using the lower THF:GPTMS ratios of 50:1 and 25:1. Viscosity of the sol increased with time and the ideal point for mixing Pots 1 and 2 was when the viscosity increased to the point at which bubbles began to form, which led to formation of cylinders with reproducible morphology. The time taken for the polymerisation to progress to this suitable viscosity (bubble formation), was not reproducible. It was not affected by variation in ambient temperature and humidity.

**Fig. 2:**
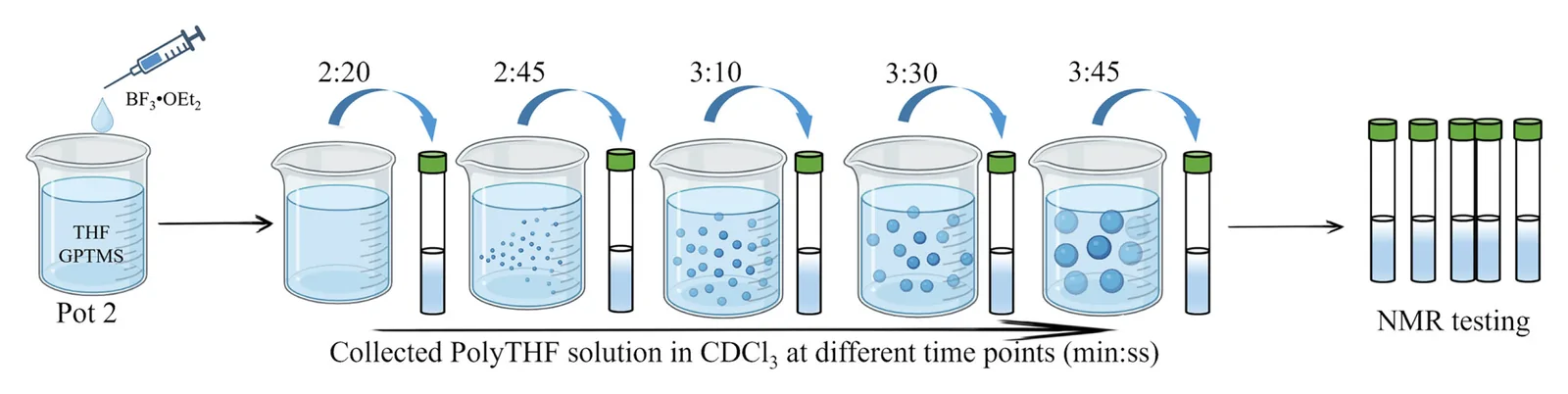
Schematic diagram of collection of ^1^H-NMR samples to check the kinetics of PolyTHF formation in a synthesis with THF:GPTMS 50:1 molar ratio. The in situ cationic ring-opening polymerisation of the organic network occurs rapidly upon the addition of BF_3_·OEt_2_ catalyst in THF/GPTMS solution (Pot 2), leading to the formation of bubbles. Subsequently, a drop of THF/GPTMS solution was collected in an NMR tube containing CDCl_3_ to terminate the polymerisation. This was repeated at specific time points after catalyst addition (min:ss), until they became too viscous to collect. The samples were then ready for NMR testing

**Fig. 3:**
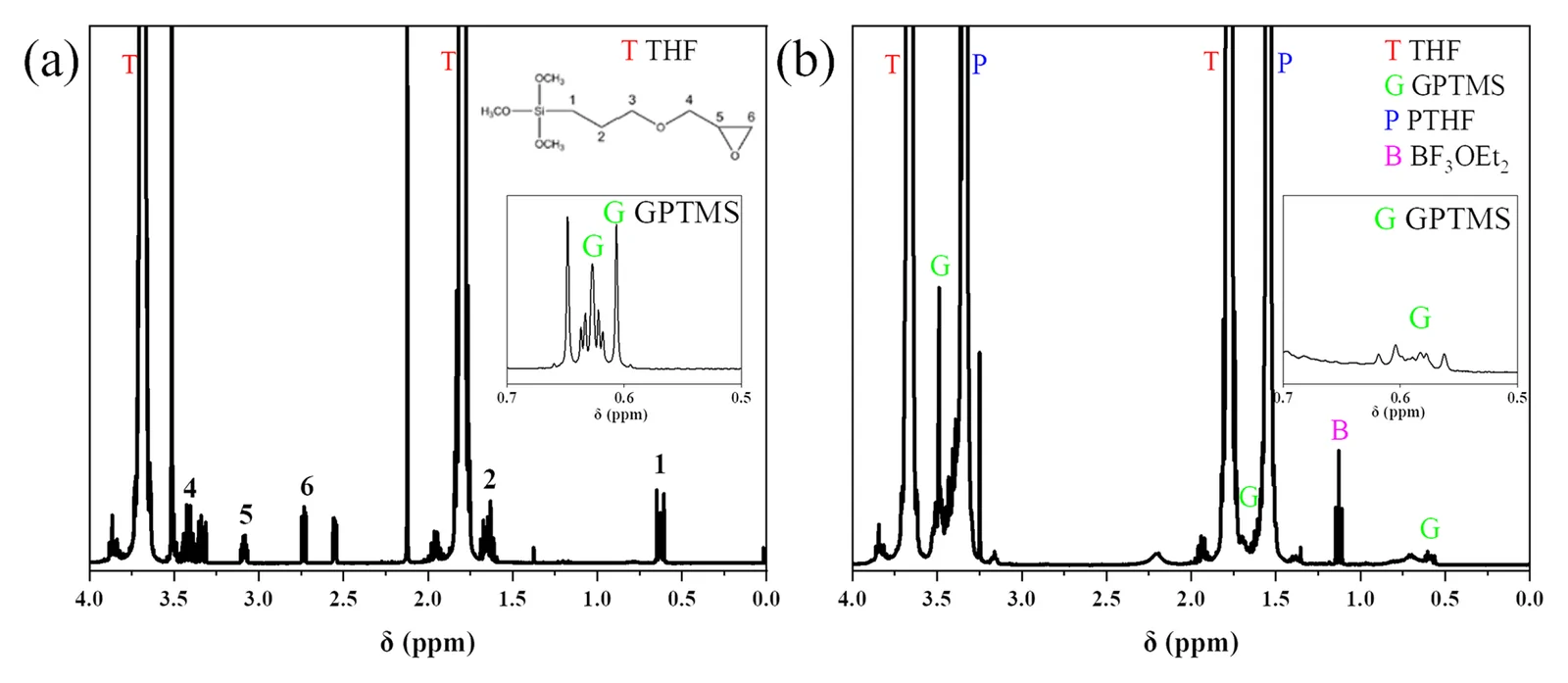
^1^H NMR spectra of Pot 2: **a** THF and GPTMS stirred for 15 min without added BF_3_·OEt_2_ catalyst, with GPTMS chemical structure labelled with associated NMR peaks; **b** THF and GPTMS, 3 min and 45 s after addition of BF_3_·OEt_2_ catalyst. Molecular weight calculations of PolyTHF coupling reaction with GPTMS were calculated using integration of the area under the GPTMS characteristic peak at 0.56–0.66 ppm (highlighted in figure) and PolyTHF characteristic peak at 1.54 ppm

The hybrid preparation used a two-pot synthesis method. Samples for ^1^H solution NMR were collected from Pot 2 (in situ CROP of the organic network) following the steps depicted in Fig. 2 to test the synthesis kinetics. The THF:GPTMS ratio of 50:1 was selected since it was the ratio giving the most successful hybrid monoliths. A theoretical molar mass (M_n_) calculation can determine the trends of further synthesis. ^1^H solution NMR was used to follow the Pot 2 reaction and to calculate M_n_ of the organic formed during in situ CROP as time progressed after BF_3_·OEt_2_ catalyst addition. In ^1^H NMR spectra, the area under each resonance signal is proportional to the molar concentration of the protons being analysed [31]. Figure 3a shows ^1^H solution NMR of Pot 2 before the addition of BF_3_·OEt_2_, i.e. THF and GPTMS mixing without any catalyst. Figure 3b shows a representative ^1^H NMR spectrum of the Pot 2 reagents (THF and GPTMS) 3 min and 45 s after adding the BF_3_·OEt_2_ catalyst. The M_n_ of the product of the PolyTHF coupling reaction with GPTMS can be calculated using Eq. (1):

(1) $$M_{n}=Mw_{GPTMS}+N\times Mw_{THF}$$

where Mw_GPTMS_ is the molar mass of GPTMS initiator (236.34 g/mol) and N is the number of repeating units of THF and Mw_THF_ is the molar mass of a THF unit (72.11 g/mol). N for PolyTHF-GPTMS was calculated according to Eq. (2) for different time points up to 3 min and 45 s after adding the BF_3_·OEt_2_ catalyst [31, 32]:

(2) $$\frac{\;Peak\;area\;of\;repeating\;units\;}{\;Number\;of\;protons\;in\;repeating\;units\;(N)}=\frac{\;Peak\;area\;of\;end\;groups\;}{\;Number\;of\;protons\;in\;end\;groups\;}$$

Considering Fig. 3b as example, the peak at δ = 0.56–0.66 ppm was identified as the GPTMS peak and PolyTHF peak as δ = 1.54 ppm. The choice of the peak at a lower ppm position for measurement of a polymer’s M_n_ is due to its higher sensitivity, and independent peak presentation (without overlap) allows for greater accuracy [33].

**Fig. 4:**
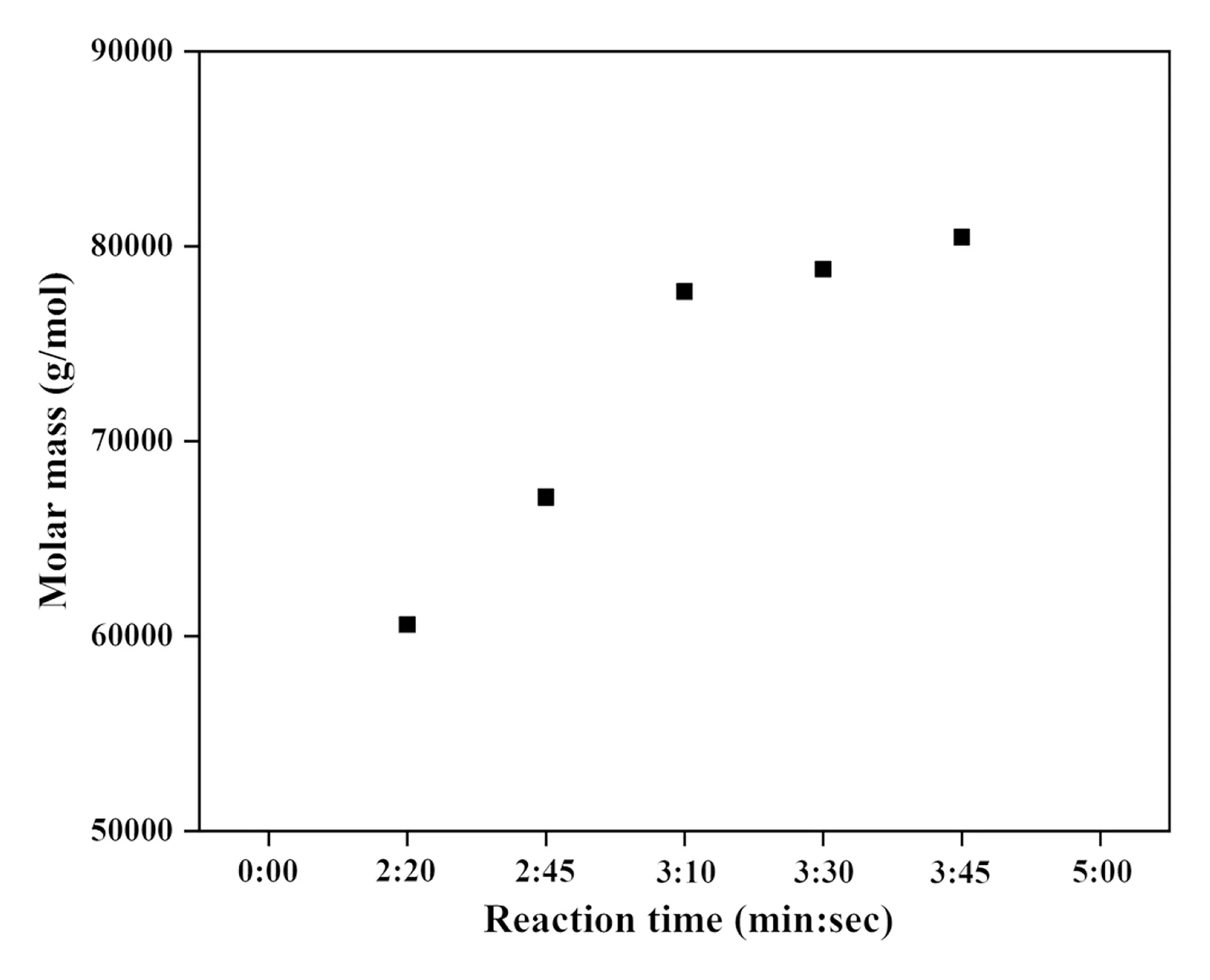
Hybrid material with THF:GPTMS molar ratio of 50:1 was selected for molecular weight evaluation. The molar mass of PolyTHF formed by the coupling reaction with GPTMS during the in situ CROP, determined from the NMR spectra as a function of mixing time after addition of BF_3_·OEt_2_ catalyst. The solution became too viscous to do the test after 5 min of mixing

For this example, the repeating unit was represented by the PolyTHF peak as δ = 1.54 ppm, while the end group was the -CH_2_ from GPTMS at 0.56–0.66 ppm (identified as 1 in Fig. 3a). The area of the repeating units and of the end groups can be obtained by integrating the area under the corresponding resonance peaks from ^1^H-NMR data and then substituting it into Eq. (2) [31]. Once the number of protons in repeating units (N) was obtained the results were input into Eq. 1 to obtain the final M_n_ of the organic net-work. For instance, N measured after 3 min and 45 s of polymerisation was estimated to be 920, giving a M_n_ of 67,000 g/mol. Figure 4 shows M_n_ as a function of time after BF_3_·OEt_2_ addition. Immediately after catalyst addition, the sol was transparent. After 2 min and 20 s, small bubbles began to form; after 3 min and 10 s, the number of bubbles filled the entire solution and after 5 min the viscosity was too high for sample collection. M_n_ plays a crucial role in this experiment because the formation of bubbles occurs when the M_n_ reached approximately 60,000 g/mol, and it rapidly increased to about 80,000 g/mol, completing the reaction in about one minute. When the molecular weight exceeded 80,000 g/mol, the organic component became too viscous to proceed with the follow-up reaction for hybrid synthesis. Therefore, we can infer that the optimal M_n_ range for synthesis lies between 60,000–80,000 g/mol. This experiment was conducted under the conditions of a 50:1 THF:GPTMS ratio. Under these conditions, there was a high success rate for forming uncracked monoliths, and the reactions generated during the synthesis were all similar. Other compositions may produce different outcomes in terms of M_n_ values due to kinetics variation, but exhibited similar trend of increasing M_n_ with increasing time from catalyst addition [34].

**Fig. 5:**
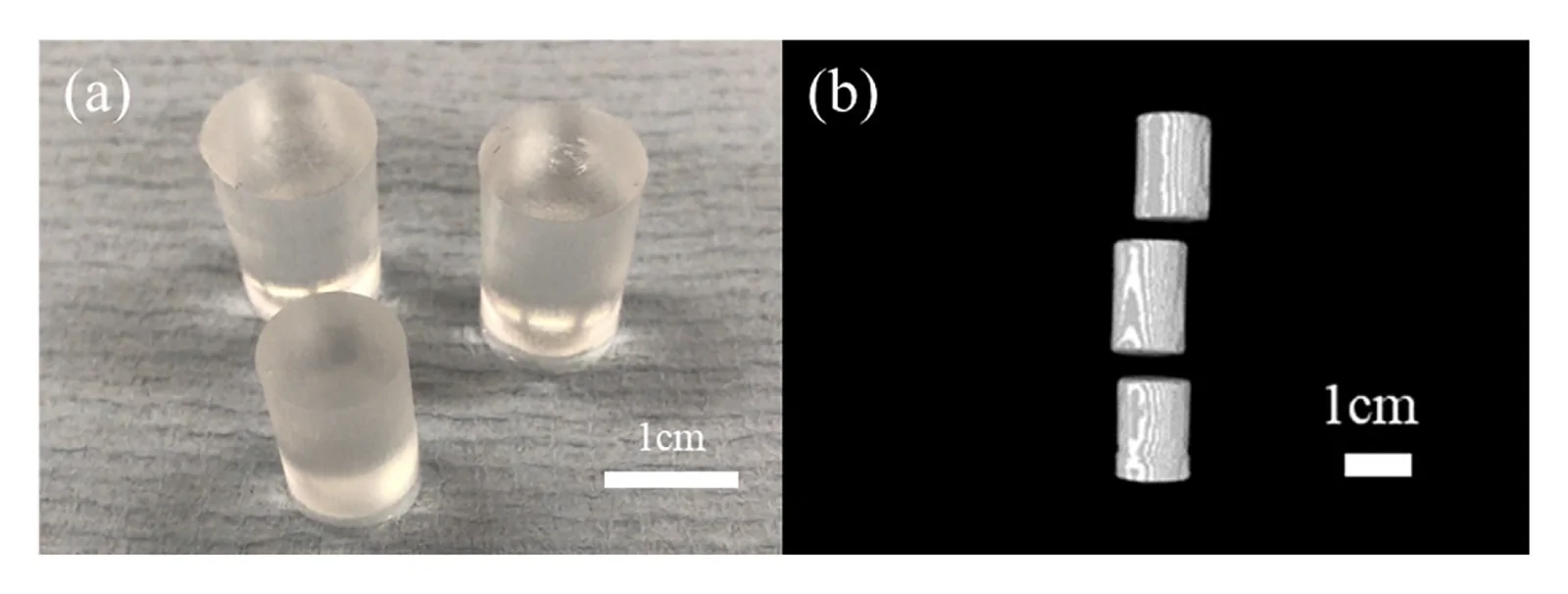
**a** Photographs of 50FD cylindrical samples; **b** 3D X-ray microtomography images of the 50FD cylindrical monoliths used for volume shrinkage calculations

Figure 5a shows photographs of examples of successful 50FD hybrid cylinders. It is worth noting that there is no significant difference in appearance between the OD and FD materials. To understand the shrinkage factor of the monoliths, dimensions of cylindrical samples were measured by digital Vernier callipers. Volumes were also calculated from μCT images (Fig. 5b) using computational script calculation and the results have been listed in Table 2. The measured volume shrinkages (78.4–87.0%) were similar to computational volume shrinkages (77.3–87.3%). Final cylinder diameters after drying ranged from 59.9 to 65.5% of the original mould diameter, demonstrating a lower shrinkage compared to the height, which decreased to values ranging from 54.7 to 30.9% of the original height (25 mm); this suggests that shrinkage was not isotropic, because despite being observed in all directions, it was notably more pronounced vertically [35]. Cylinders with the highest THF:GPTMS ratio (100:1) underwent the most volume shrinkage of up to 87.3%. No significant difference was noticed among OD and FD samples made with the same THF:GPTMS ratio, meaning that the drying processes had little effect on shrinkage. THF acts as both a reactant and solvent during polymerisation. When the amount of coupling agent GPTMS was fixed, increasing the amount of THF hindered the progression of the polymerisation. Increasing the THF volume, while GTPMS remained constant, reduced the rate of increase in the viscosity of the sol (rate of gelation) and made it difficult to observe bubbles, even if the reaction was prolonged. The results suggest that for high THF:GPTMS ratios (i.e., 100:1 and 75:1), there is a proportion of THF that acts as solvent without polymerising, resulting in a slower polymerisation due to the dilution of the solution and more solvent to evaporate which consequently produces a greater shrinkage. In these cases, to have a good condensation of the network a very slow drying process is required, to avoid cracking or deformation of the samples. As detailed in Fig. 1, this was not achieved with THF:GPTMS of 100:1, where distorted or hollow cylinders were always produced, while with THF:GPTMS of 75:1 only 4 batches out of 16 gave homogeneous samples. Using a THF:GPTMS 50:1 ratio represents the optimal balance between THF that participates in the reaction and excess THF that acts purely as solvent, in order to be able to control the polymerisation (i.e., adding Pot 1 before Pot 2 is completely gelled) but also have good quality and crack-free samples with a reasonable shrinkage. When THF:GPTMS ratio of 25:1 was used, crack-free and regular samples were obtained but there was little excess THF to act as solvent and a rapid polymerisation of the organic solution (around 1 min) was observed. This made it harder to handle the solutions and very fast action was required to add the hydrolysed TEOS before the organic solution became too viscous.

### 3.2 Hybrid compositional analysis

**Fig. 6:**
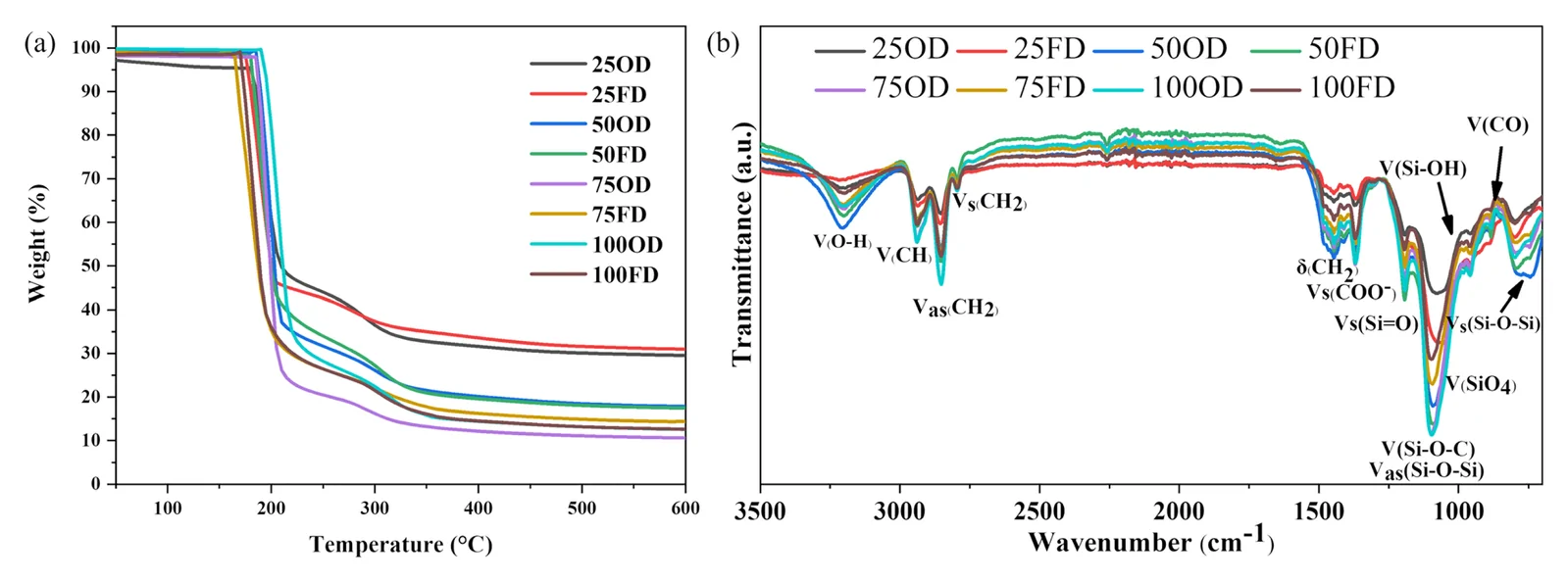
**a** TGA analysis for quantification of the relative inorganic and organic content in SiO_2_-PolyTHF hybrids. **b** FTIR spectra of SiO_2_-PolyTHF hybrids. The legend codes represent molecular ratio of THF:GPTMS according to Table 1 and the drying method (oven dried (OD) vs freeze dried (FD))

TGA analysis was used to determine the I/O ratio of the monoliths after drying (Fig. 6a and Table 3). For the 25OD sample, approximately 4 wt% of residual water evaporation occurred up to 100 °C and, as the boiling point of GPTMS is approximately 120 °C, about 0.5 wt% mass was lost from 120 to 180 °C [36]. The majority of weight loss occurred from 180 °C until 350 °C, corresponding to the thermal degradation of the organic network. When GPTMS is thermally decomposed, the side chains of the organic network are decomposed until the silica-rich domains remain. The weight loss slowed around 210 °C because the remaining organic network is protected by the silica network. Eventually, at around 350 °C, all of the organic matter, formed by PolyTHF and the organic chain of GPTMS, was decomposed, leaving only the inorganic residue [37]. Residual silica contents for each set of monoliths were 31.7% (25OD), 31.2% (25FD), 18.0% (50OD), 17.1% (50FD), 13.1%(75OD), 15.3% (75FD), 12.4% (100OD), and 13.8% (100FD). Theoretically, if all the THF put into the system polymerised into PolyTHF, 50OD (18.0%), 75OD (13.1%) and 100OD (12.4%) should have a two-, three and four-fold less inorganic part relative to the 25OD (31.7%) sample, respectively. However, the TGA results indicated only a 1.8, 2.4 and 2.5-fold difference in the inorganic content, respectively. A similar trend was observed for freeze-dried samples, with a 1.8, 2.0 and 2.3- fold difference in the inorganic part of 50FD, 75FD and 100FD to 25FD, respectively. This confirmed that when more THF was added, not all of it polymerised into PolyTHF, some remained as solvent during synthesis and evaporated during drying. The deviation from the expectations was most evident for the samples with THF/GPTMS of 75 and 100. It is hypothesised that the rate of THF polymerisation was fastest soon after BF_3_ ∙ OEt_2_ addition, then it decreased and progressed slowly over time until all the initiator reacted or the reaction was quenched by the addition of the inorganic (TEOS-based) solution. With a THF/GPTMS molar ratio of 50, most of the THF polymerised before the addition of the inorganic solution, and just a small portion was in excess. For higher ratios (75 and 100), a significant portion of excess THF did not polymerise (solvent in excess compared to reactants), and evaporated during drying, leading to a lower increase in the organic content than expected. This is also in agreement with the shrinkage values that suggested a higher THF evaporation the more THF was put in the system.

FTIR spectra (Fig. 6b) were similar for all samples. The absorption of the Si-O-Si symmetric vibration was visible at around 800 cm^−1^, 1000–1100 cm^−1^, and the Si-OH stretch at around 960–980 cm^−1^; the broad O-H stretch centred at 3200 cm^−1^ is attributable to silanol (Si-OH) groups and absorbed water. Other organic absorption bands like COO^-^ at around 1370 cm^−1^, CH_2_ bends at around 1450, 2800, and 2850 cm^−1^ were visible [38]. The Si-OH absorption band in the range of 960–980 cm^−1^ decreased in relative intensity as silica content decreased, and there was also a shift in the Si-O stretch band from 1079 cm^−1^ to 1042 cm^−1^, attributed to more oxygen-containing structure from H-Si-O-Si-H to H-Si-O-Si(O)-H in samples with higher silica content (25OD and 25FD). In samples with lower THF amount (GPTMS and catalyst are in the same concentrations for all samples), rapid polymerisation was observed.

### 3.3 Mechanical properties

**Fig. 7:**
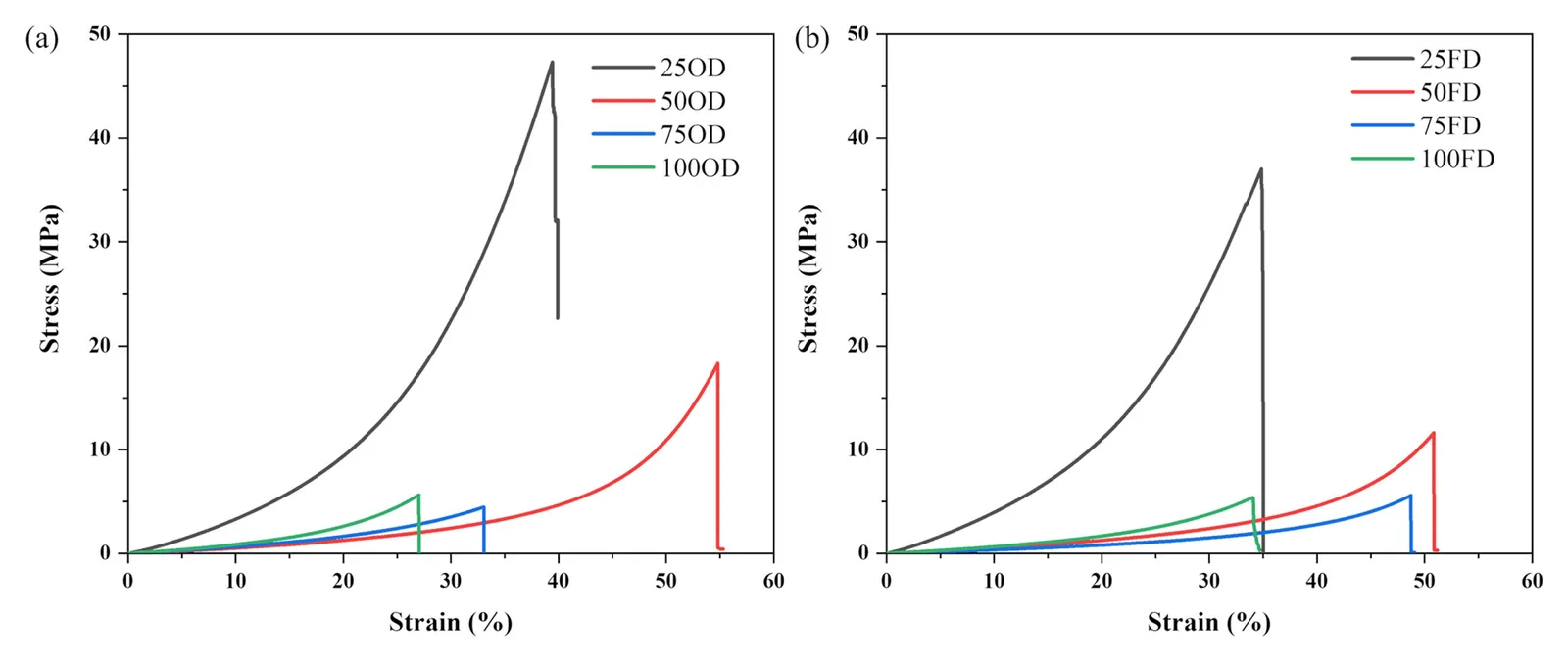
Representative stress-strain curves for SiO_2_-PolyTHF hybrid cylinders following: **a** oven drying (OD); **b** freeze drying (FD)

A combination of high strength and elastic ductility is the primary objective for monolithic hybrids. Representative stressstrain curves are shown in Fig. 7. In the compressive strength testing, a J-curve mode of deformation is observed; at low stress, materials with a high organic content exhibited significant elastomeric deformation but stress did not increase significantly until fracture occurred. Table 3 reports the values of strain and stress at failure of the different OD and FD hybrid compositions. Cylinders with higher silica content, 25OD cylinders (31.7% of silica), had highest compressive strength of 44.1 ± 2.9 MPa at a strain of 38.5 ± 0.7%. Lower strength was seen for cylinders with low silica content, with 75OD (13% silica) failing at 4.2 ± 0.2 MPa, at the lowest strain to failure of 34.2 ± 2.6%, indicating poor mechanical properties and quality. The 50OD and 50FD samples demonstrated greater strain to failure (54.5 ± 0.5 and 50.4 ± 0.2% with compression strength of 17.7 ± 0.8 and 11.1 ± 0.3 MPa), at a higher strength than the 100OD and 100FD cylinders (4.6 ± 0.3 and 5.3 ± 0.0 MPa), but at lower strength than the 25OD and 25FD cylinders (44.1 ± 2.9 and 30.5 ± 5.9 MPa). As expected, considering the high silica content, the monoliths with 31.7% silica (25OD and 25FD) failed with glass-like brittle fracture, shattering into pieces. In contrast, the 50OD and 50FD samples exhibited vertical cracks and maintained their shape without shattering. In summary, samples with 50OD and 50FD exhibited a better balance between stress and strain, making them suitable for soft tissue replacement, such as cartilage. Previous studies have indicated that physiological activities apply cyclic stresses to articular cartilage in the range of 1–18 MPa [39]. On the other hand, the stronger 25OD and 25FD samples could potentially be used to produce porous scaffolds in higher loading tissues, such as trabecular bone (2–12 MPa) [40].

**Fig. 8:**
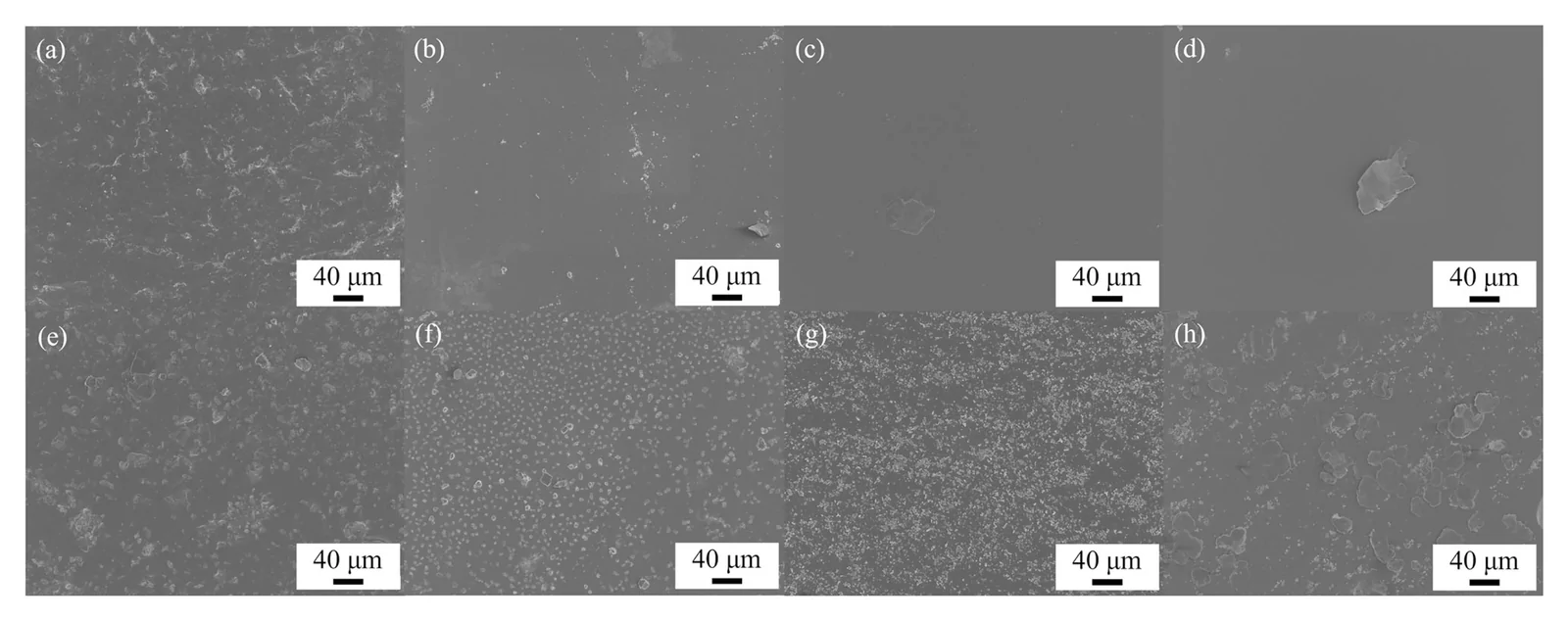
SEM images of SiO_2_-PolyTHF hybrid surface morphology **a** 25OD, **b** 50OD, **c** 75OD, **d** 100OD, **e** 25FD, **f** 50FD, **g** 75FD, and **h** 100FD

**Fig. 9:**
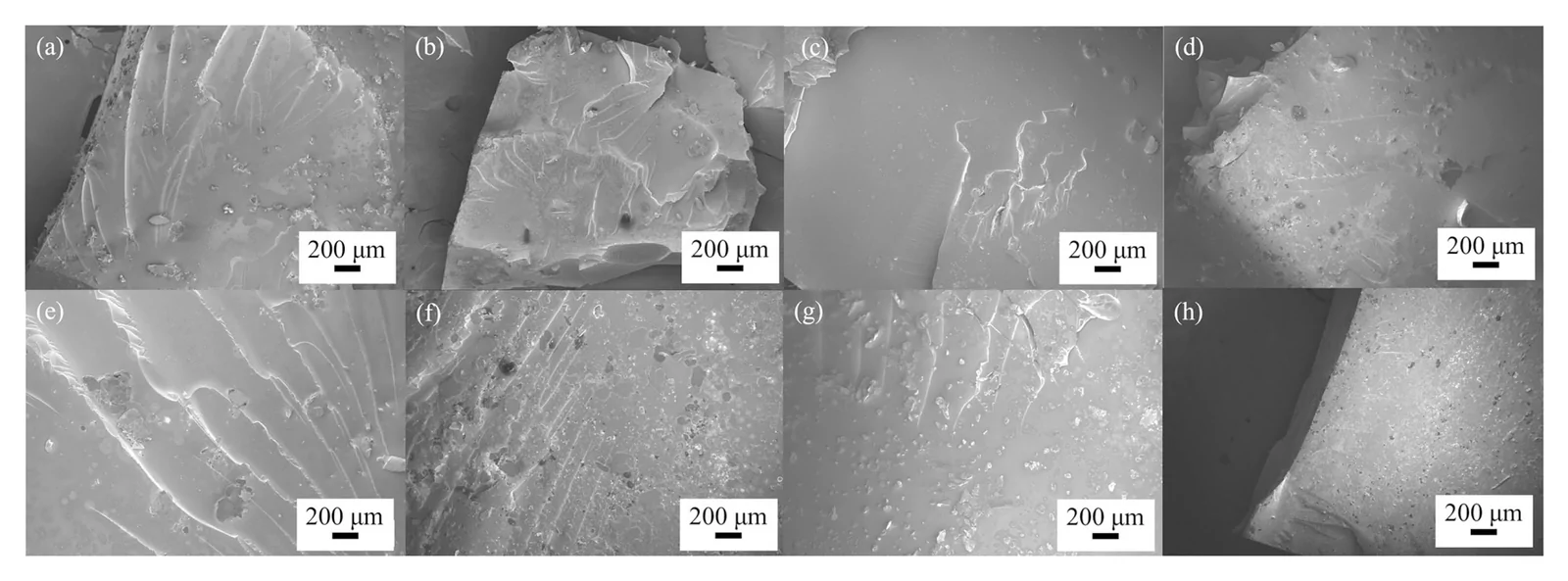
SEM images of SiO_2_-PolyTHF hybrid fracture surfaces after compression test to failure: **a** 25OD, **b** 50OD, **c** 75OD, **d** 100FD, **e** 25FD, **f** 50FD, **g** 75FD, and **h** 100FD

**Fig. 10:**
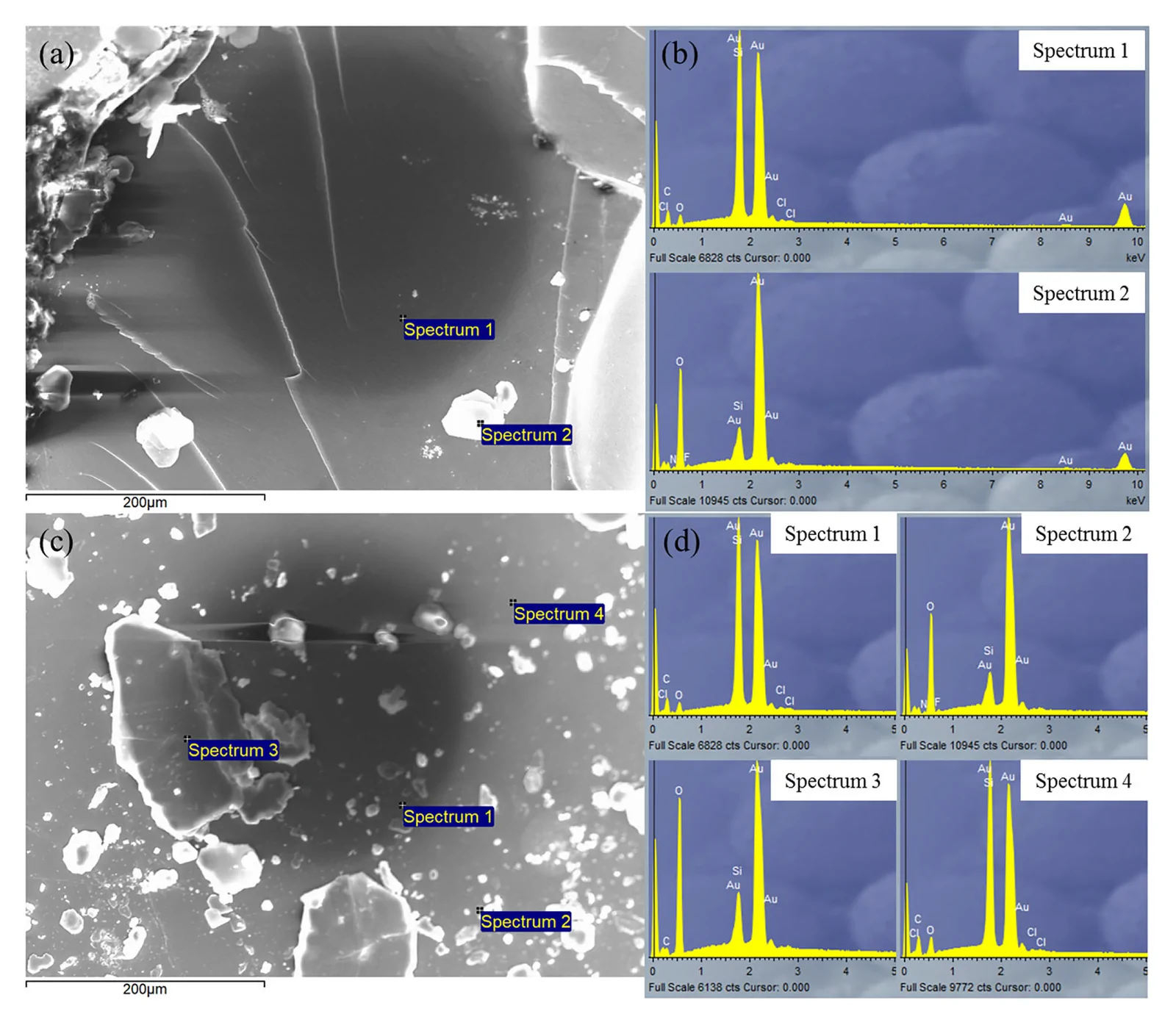
**a** SEM image of the surface of 50OD sample; **b** EDS spectra from (**a**): spectrum 1 from the flat surface, spectrum 2 from precipitate; **c** SEM image of the surface of 50FD sample; **d** EDS spectra from (**b**): spectrum 1 and 4 are obtained from the flat surface, while spectra 2 and 3 are acquired from the precipitates. It is evident that the Si peak intensity is notably stronger on the flat surface compared to the precipitates

The cylinders with a THF:GPTMS ratio of 25:1 and 50:1 that underwent FD exhibited lower compressive strength and strain compared to the corresponding OD cylinders (Table 3). However, for THF:GPTMS ratios of 75:1 and 100:1, the compressive strength of FD samples showed only a slight increase to the one of OD samples, while the strain exhibited a noticeable increase. SEM images (Fig. 8) showed that the FD process may have caused some phase separation. SEM images illustrated that the surface morphology of all as produced samples was approximately flat without obvious cracks and defects, but the FD samples had irregular island-like phase separation (Fig. 8). SEM images of the fracture surfaces after compression testing are shown in Fig. 9. No obvious structural cracks were found in any samples. Fracture patterns formed on the high silica content sample surfaces were more densely distributed on the FD sample surfaces. EDS analysis was used to indicate silicon content of 50OD and 50FD (Fig. 10), with spectra from the flat fracture surfaces and the island-like phase separation. The flat surface produced a stronger silicon signal compared with island-like phase separation, which indicated the phase separation had higher organic content rather than being higher inorganic phase separation. In Fig. 9, it can also be observed that for THF:GPTMS ratios of 75:1 and 100:1, the organic phase separation became significantly larger compared to the dense and small phase of the 50OD and 50FD samples. While a larger phase separation can act as a structural reinforcement barrier, its primarily organic composition limits its effectiveness in improving compressive strength. Conversely, it provides the structure with better strain capacity. This is evident from the brittle fracture cracks, which are shorter and less dense compared to those in the 25OD and 25FD samples. As a result, the compressive strength did not show a significant increase, but the strain exhibited a notable improvement. Meanwhile, in the structures of 25FD and 50FD, the smaller organic phase separation created structural defects, leading to a certain degree of decline in mechanical properties. The difference in phase separation is likely due to OD and FD samples being prepared in different drying processes. While OD process is carried out at constant 40 °C, FD samples were aged at 40 °C oven for 7 days then moved to −80 °C freezer to cool the sample before the −93 °C FD process and then moved back at 40 °C. This cooling stage may potentially cause phase separation on unreacted or semi-reacted THF and consequently observed the organic-rich phase separation in the SEM images. These phase separations become potential stress concentration points in the structure during compression.

### 3.4 Behaviour in wet environment

**Fig. 11:**
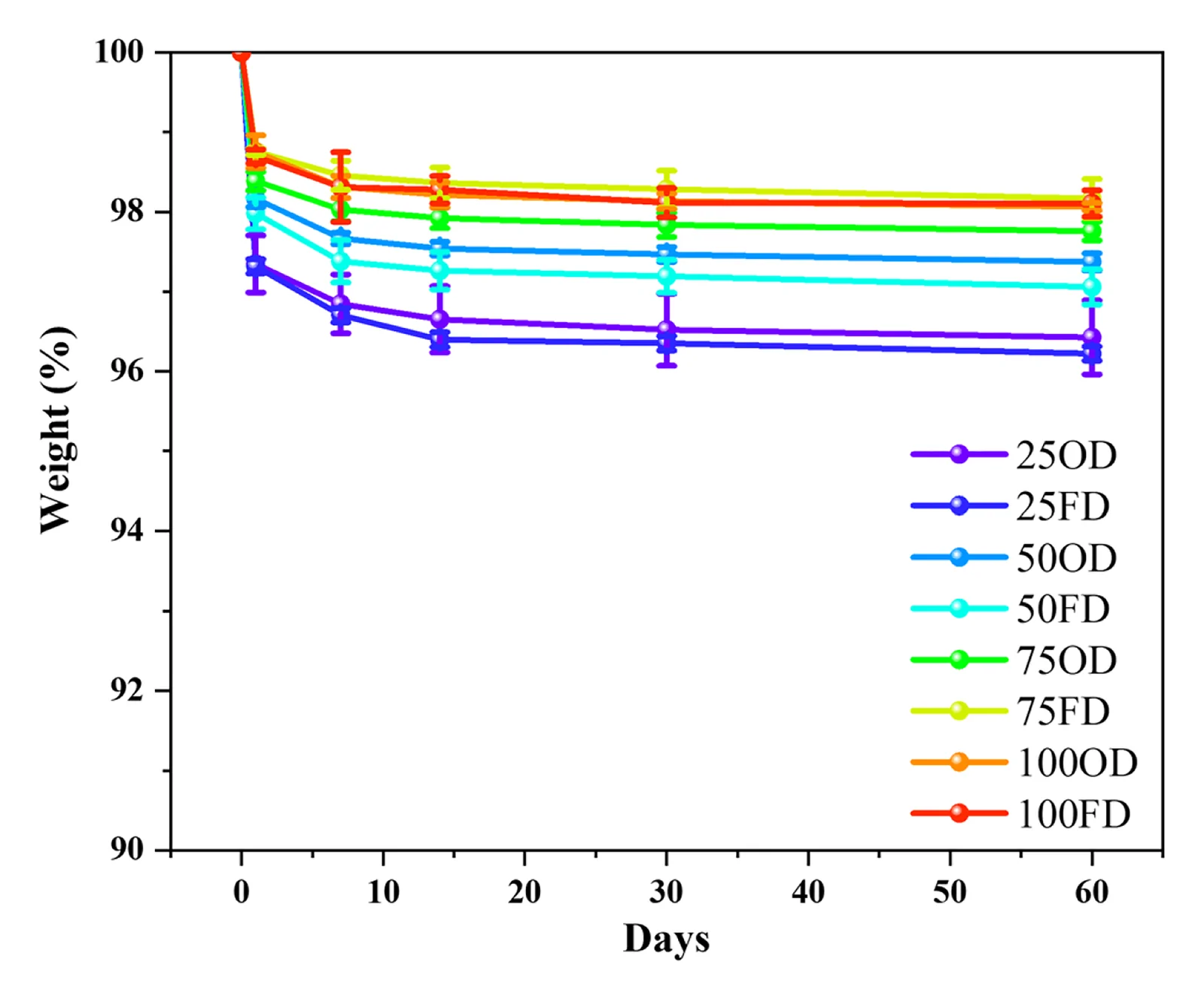
Mass before and after soaking SiO_2_-PolyTHF hybrids in PBS at 37 °C, agitated at 120 rpm. The samples are named by the molecular ratio of THF:GPTMS and the drying methods (oven drying (OD) and freeze drying (FD))

Mass of the samples was recorded before and after soaking in SBF for 60 days and the results are shown in Fig. 11. Nearly 2.7% of weight loss occurred on the first day in the high silica content samples (25OD and 25FD), with an additional decrease of about 0.5% after 7 days. However, the lower silica content sample experienced a weight loss of 1.3–1.6% on the first day and less than 0.5% over the next 6 days. Mass loss may be higher for higher silica content because the silica network is hydrophilic and can degrade in water at regions of low network connectivity. The mass remained stable after 14 days of the test, with weight loss less than 0.1% in each composition, confirming the non-biodegradable nature of this hybrid system.

**Fig. 12:**
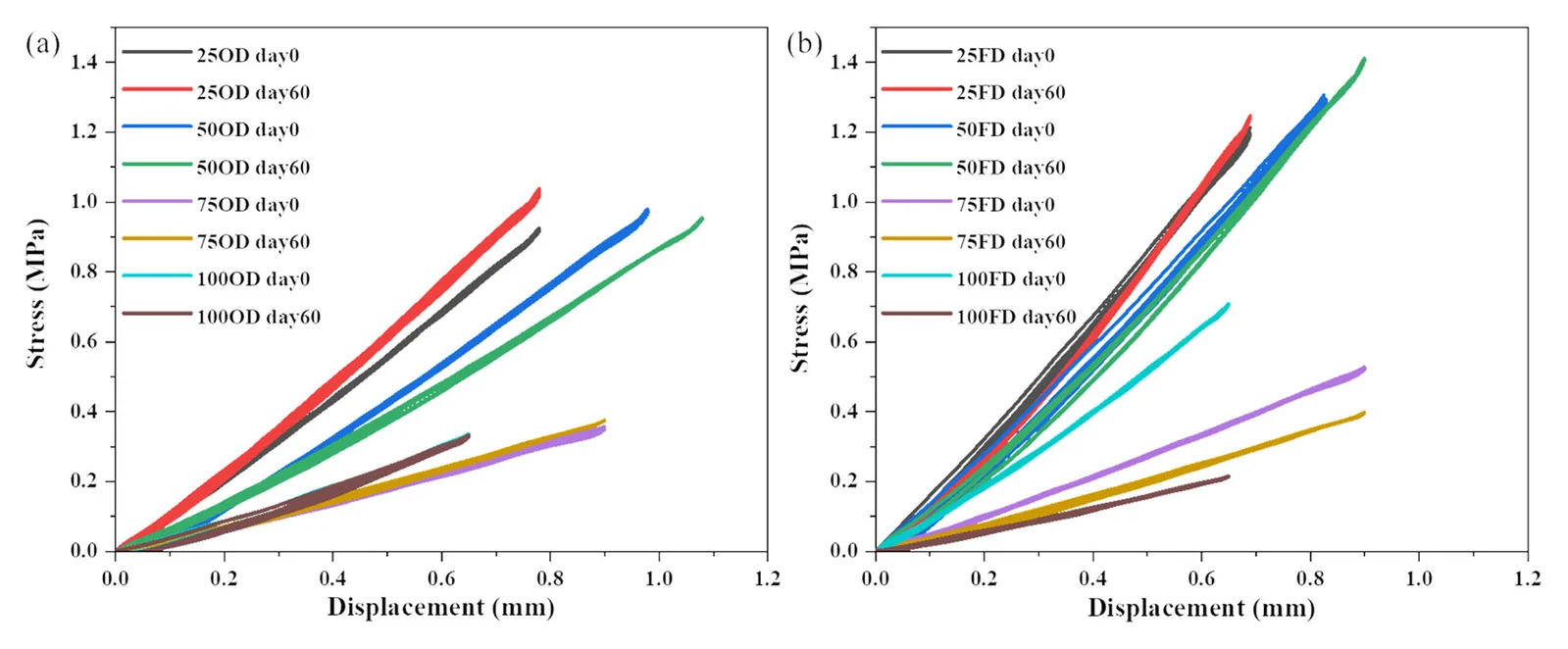
Example of cyclic compression tests of SiO_2_-PolyTHF hybrids before (day 0) and after being soaked in PBS at 37 °C and 120 rpm for 60 days (day 60): **a** OD samples; **b** FD samples. The strain employed for the cyclic loading test equalled 20% of the deformation observed at failure during the compression test. For each sample at a specific time point, curves collected every 100 cycles up to the maximum of 1000 are plotted: these curves overlap, demonstrating the stability of the mechanical properties over the course of the test

Cyclic compression testing was carried out on cylindrical hybrids before and after soaking in PBS at different time points, up to 60 days. The samples experienced 1000 cycles in compression test before being soaked (day 0) in PBS. Subsequently, they were subjected to 1000 cycles at 1 cycle/s at each time point following 1, 7, 14, 30, and 60 days of soaking, and the tests were conducted under wet conditions. The cyclic test curves for the OD and FD samples at day 0 and day 60 are shown in Fig. 12a, b, respectively, with corresponding values of stress and strain presented in Table 4. The cyclic curves showed no hysteresis at day 0 and the appearance of the material did not change even after 1000 cycle compression loading in OD and FD samples. Although the FD samples demonstrated lower compression strength, the cyclic patterns remained a regular elastic deformation behaviour after 60 days soaking. The cyclic test results for the 25OD sample showed a slight increase in strength, which may be attributed to some water entering the structure. However, the other OD samples did not exhibit noticeable change in stress after 60 days of soaking in PBS. In contrast, the 25FD and 50FD samples demonstrated a slight increase in strength also related to water absorption into the structure. Meanwhile, the 75FD and, notably, the 100FD samples showed a significant decrease in stress in high THF content samples, with 100FD decreasing from 0.7 MPa to 0.21 MPa at 20% engineering strain. The possible reason is that excessive or larger areas of organic phase separation seen in the SEM images may lead to enlarged regions of elastic polymer, causing a decrease in stress at a particular strain. In general, the SiO_2_-PolyTHF hybrid monoliths exhibited excellent structural integrity and good mechanical reliability which brings many potential applications to the field of biomaterials such as cartilage replacement, joint defect filler, and intervertebral disc replacement.

From a previous study by Young et al. [23], it was indicated SiO_2_-PolyTHF system can possess excellent mechanical properties. Adjusting the proportion of SiO_2_ in the overall structure from 6 to 32 wt% SiO_2_ under a fixed THF:GPTMS ratio of 100:1 achieved gradient mechanical properties. The overall compression strength of the material was enhanced from 5.1 MPa to 32.7 MPa [23]. This means the hybrid system could be tailored to match the mechanical properties of cartilage. Meanwhile, the hybrid material can be made into different shapes; when mixing organic and inorganic components, this material increases the overall solution viscosity, allowing for casting within a certain timeframe. Therefore, this material can potentially serve as an excellent ink for direct ink write 3D printing material [20, 41]. This study investigates the adjustment of different THF monomer ratios. Although altering the THF monomer ratio indirectly changes the I/O ratio, varying the concentration of GTPMS during the polymerisation process can lead to a higher initial initiator concentration, allowing the entire structure to better facilitate the concentration of growing centres for polymerisation [42]. Therefore, the results of this experiment show that the I/O ratio can also ensure the structural integrity of the material and reduce the occurrence of fractures, thereby improving its mechanical properties.

## 4 Conclusions

This research conducted a comprehensive study on SiO_2_-PolyTHF hybrid monolith synthesis by control of I/O ratios through variation of THF content with respect to GPTMS and testing of different drying processes. This hybrid material inherits a synergy of properties from the inorganic and organic components. The I/O ratio plays a crucial role in enhancing monolith compressive strength. By adjusting the I/O ratio, the hybrids showed a compressive strength that can be tailored from 4 to 44 MPa, and they can with-stand up to 54% strain, making it suitable for various applications, without hysteresis under cyclic loading. The hybrid was synthesised by in situ CROP to form a hybrid of PolyTHF and silica, with covalent coupling via GPTMS to give excellent structural integrity. OD produced better material stability in mechanical testing and less phase separation than FD. NMR results suggested that approximate molecular weight of Poly-THF changed rapidly during the synthesis, and a complete hybrid monolith structure can be obtained by mixing two pots at an appropriate stage in the polymerisation. These results show how silica-based hybrids can be produced with good mechanical properties in the form of large hybrid monoliths.

## Figures and Tables

**Table 1:** Reagent quantities for SiO_2_-PolyTHF hybrid synthesis, where A refers to the moles of GPTMS and B refers to the moles of TEOS

Reagent	Molar ratio
GPTMS	A
BF_3_·OEt_2_	0.25A
THF	25A, 50A, 75A, 100A
TEOS	B = 2.5A
H_2_O	C = 3A + 4B
HCl (1 M)	1/3 volume of C

The organic ratios are controlled by the THF/GPTMS molar ratios which are from 25:1 to 100:1

**Table 2:** Shrinkage behaviour of SiO_2_-PolyTHF hybrid monoliths

Sample	25OD	25FD	50OD	50FD	75OD	75FD	100OD	100FD
Volume shrinkage (%)								
Measurement	82.0 ± 1.2	82.0 ± 0.6	78.4 ± 0.2	79.5 ± 0.1	81.9 ± 0.5	80.0 ± 1.2	87.0 ± 0.7	86.7 ± 0.5
Computational	81.2 ± 0.7	80.8 ± 1.5	77.3 ± 1.2	80.5 ± 1.2	79.1 ± 0.3	78.2 ± 0.1	87.3 ± 3.1	85.6 ± 1.3
Dimensional shrinkage to (%)								
Height	47.7 ± 3.2	49.2 ± 2.2	52.8 ± 0.5	54.7 ± 1.1	50.5 ± 2.3	46.8 ± 0.4	36.4 ± 3.4	30.9 ± 0.9
Diameter	61.3 ± 0.1	60.4 ± 0.4	62.2 ± 0.4	62.7 ± 0.4	62.8 ± 0.6	62.0 ± 0.6	59.9 ± 4.0	65.5 ± 0.8

Dimensional shrinkages were measured by vernier callipers (*n* = 3)

Volumetric shrinkage was evaluated by computational analysis of X-ray microtomography images and geometric (vernier calliper) measurement

**Table 3:** Stress and strain at fracture for hybrids with different drying techniques at each inorganic / organic (I/O) ratio

Sample	25OD	25FD	50OD	50FD	75OD	75FD	100OD	100FD
I/O ratio (%)	31.7/68.3	31.2/68.8	18.0/82.0	17.1/82.9	13.1/86.9	15.3/84.7	12.4/87.6	13.8/86.2
Strain (%)	38.5 ± 0.7	32.4 ± 2.4	54.5 ± 0.52	50.4 ± 0.2	34.2 ± 2.6	42.7 ± 5.08	31.3 ± 2.5	36.5 ± 3.9
Stress (MPa)	44.1 ± 2.9	30.5 ± 5.9	17.7 ± 0.8	11.1 ± 0.3	4.2 ± 0.2	4.7 ± 0.7	4.9 ± 0.3	5.3 ± 0.0

The samples are named by the molar ratio of THF:GPTMS (as Table 1) and the drying methods (Oven drying (OD) and freeze drying (FD)) which are 25OD, 25FD, 50OD, 50FD, 75OD, 75FD, 100OD, and 100FD (*n* = 3). The I/O wt.% were measured via TGA

**Table 4:** Values of displacement (set as 20% of displacement at failure from compression test) and stress (measured as output) for the samples tested in cyclic loading in Fig. 12

Sample	25OD Day 0	25OD Day 60	50OD Day 0	50OD Day 60	75OD Day 0	75OD Day 60	100OD Day 0	100OD Day 60
Displacement (mm) Stress (MPa)	0.77 0.91 ± 0.01	0.77 1.02 ± 0.01	0.97 0.97 ± 0.01	0.97 0.95 ± 0.01	0.90 0.35 ± 0.01	0.90 0.37 ± 0.01	0.65 0.33 ± 0.01	0.65 0.32 ± 0.01
Sample	25FD Day 0	25FD Day 60	50FD Day 0	50FD Day 60	75FD Day 0	75FD Day 60	100FD Day 0	100FD Day 60
Displacement (mm) Stress (MPa)	0.68 1.19 ± 0.01	0.68 1.24 ± 0.01	0.82 1.29 ± 0.01	0.82 1.40 ± 0.01	0.89 0.52 ± 0.01	0.89 0.40 ± 0.01	0.64 0.70 ± 0.01	0.64 0.21 ± 0.01

The stress is reported as mean ± standard deviation calculated from the values measured every 100 cycles within the 1000 cycle test (i.e., corresponding to the curves reported in the graphs in Fig.12)

## Data Availability

The data that support the findings of this study are available from the corresponding author upon reasonable request from rdm-enquiries@imperial.ac.uk.

## Highlights

- Crack-free silica-based hybrid monoliths prepared via sol-gel and cationic ring-opening polymerisation.
- Optimised SiO_2_-PolyTHF hybrid synthesis based on THF content and drying processes.
- SiO_2_-PolyTHF hybrids show superior performance under cyclic loading with inorganic-organic synergy.
